# From Food Structure to L-Cell Exposure: How Processing and Digestion Shape Endogenous GLP-1 Responses

**DOI:** 10.3390/molecules31193375

**Published:** 2026-09-23

**Authors:** Roko Šantić, Marko Kumrić, Tina Tičinović Kurir, Doris Rušić, Joško Božić

**Affiliations:** 1 Department of Pathophysiology, University of Split School of Medicine, Šoltanska 2A, 21000 Split, Croatia; roko.santic@mefst.hr (R.Š.); marko.kumric@mefst.hr (M.K.); tticinov@mefst.hr (T.T.K.); 2 Laboratory for Cardiometabolic Research, University of Split School of Medicine, Soltanska 2A, 21000 Split, Croatia; 3 Department of Endocrinology, Diabetes and Metabolic Diseases, University Hospital of Split, Spinciceva 1, 21000 Split, Croatia; 4 Department of Pharmacy, University of Split School of Medicine, Soltanska 2A, 21000 Split, Croatia; doris.rusic@mefst.hr

**Keywords:** glucagon-like peptide-1, enteroendocrine L cells, incretin hormones, food matrix, intestinal nutrient sensing, functional foods, bioaccessibility, gastric emptying, dipeptidyl peptidase-4, postprandial metabolism

## Abstract

Interest in using foods to stimulate endogenous glucagon-like peptide-1 (GLP-1) has outpaced evidence that transient postprandial increases translate into meaningful physiological outcomes. This review critically examines how food processing and matrix architecture interact with digestion and microbial metabolism to determine the identity, timing, intestinal location, and epithelial accessibility of GLP-1-stimulating ligands. PubMed/MEDLINE, Web of Science Core Collection, BIOSIS Citation Index, and Scopus were searched for the relevant literature. Evidence from cellular systems, organoids, perfused intestines, animal models, and human studies indicates that sugars, amino acids, peptides, fatty acids, monoacylglycerols, bile acids, and microbial metabolites engage distinct nutrient-sensing pathways. However, GLP-1 responses depend not only on chemical composition but also on cellular integrity, particle size, viscosity, starch organization, emulsion structure, protein processing, fermentation, gastric emptying, and the site of intestinal release. These factors can modify the magnitude and kinetics of GLP-1 exposure, although effects vary considerably with material, dose, comparator, population, and experimental model. Whey-containing preloads provide the most consistent acute human evidence, whereas findings for fibres, emulsions, phytochemicals, fermented foods, and controlled-release approaches remain heterogeneous. Importantly, increases in circulating GLP-1 do not consistently coincide with reduced appetite or energy intake, improved glycaemia, or sustained changes in body weight. Food-induced GLP-1 secretion, reduced degradation through dipeptidyl peptidase-4 inhibition, and direct GLP-1 receptor agonism therefore represent distinct biological mechanisms. Overall, food structure can modestly and context-dependently modulate endogenous GLP-1 exposure, but current evidence does not support physiological or therapeutic equivalence with pharmacological GLP-1 receptor agonists.

## 1. Introduction

Glucagon-like peptide-1 (GLP-1) is an incretin peptide released after nutrient ingestion. It enhances glucose-dependent insulin secretion, suppresses glucagon during hyperglycaemia, slows gastric emptying, and contributes to satiation through vagal and central pathways [1,2,3,4]. These actions make endogenous GLP-1 relevant to postprandial glycaemic control and short-term appetite regulation and provide a rationale for nutritional strategies that modulate its secretion. Broad reviews already cover diet and GLP-1 secretion [5,6], strategies to slow digestion [7], specific fibres [8], and food proteins and hydrolysates [9]. Evidence for fibre interventions nevertheless remains heterogeneous across dose, population, matrix, and study design [8]. Less clear is how food structure and digestion determine exposure of intestinal sensors and how modest endogenous responses should be interpreted clinically. Such responses should not be equated with GLP-1 receptor agonist therapy [10]. Native GLP-1 is released transiently and rapidly inactivated by DPP-4, whereas GLP-1 receptor agonists are designed to produce prolonged systemic receptor exposure [11,12,13]. Dietary stimulation should therefore be interpreted as a modulation of meal physiology rather than nutritional equivalence to pharmacological therapy.

A recent starch-focused review further linked multiscale starch structure, digestion rate, distal substrate delivery, and GLP-1-oriented functional-food design [14]. The present review extends beyond starch by comparing carbohydrate, protein, lipid, bile-acid, and microbial routes and by separating secretion from downstream clinical outcomes.

A 2026 conceptual review proposed that disruption of the food matrix by ultra-processing may accelerate oral processing and digestion, shift nutrient absorption proximally, and attenuate distal GLP-1/PYY signalling [15]. The matrix concept alone is therefore not novel. A remaining question for food chemistry is how chemically resolved digestion products reach sensors on the relevant epithelial surface and how findings from different models can be interpreted alongside assay performance and human outcomes. Enteroendocrine cells are exposed to a changing luminal mixture of sugars, peptides, amino acids, fatty acids, monoacylglycerols, bile acid species, and fermentation products. Molecular architecture and supramolecular assembly shape that mixture. Processing alters cell integrity, particle size, crystallinity, aggregation, rheology, and interfacial area, while disintegration, hydrolysis, absorption, controlled delivery, and microbial conversion further determine ligand concentration and location. Several nutrient-sensing pathways are well characterised at the cellular level [16], but their integration with food structure and digestion kinetics remains incomplete.

Human matrix studies illustrate the problem across several food classes. In a chickpea experiment with interventions matched for composition, cell disruption shifted glucose, GIP, and GLP-1 toward an earlier postprandial response, whereas intact cells delayed nutrient release and were accompanied by more prolonged GLP-1 and PYY [17]. In oat products, changes in viscosity or physical form altered gastric emptying and glycaemia without a consistent increase in GLP-1 [18,19]. In the factorial emulsion study, a liquid-lipid, acid-stable emulsion increased the 6 h incremental area under the plasma GLP-1 concentration–time curve by 38% versus the solid-lipid acid-unstable formulation and by 76% versus the liquid-lipid acid-unstable formulation [20]. These studies indicate that the timing and regional distribution of digestion products can matter as much as nutrient identity or peak GLP-1 concentration. Most, however, inferred rather than directly measured regional ligand exposure.

Accordingly, this review aims to critically integrate evidence on how food composition, multiscale matrix structure, processing operations, gastrointestinal digestion, regional intestinal delivery, and microbial metabolism determine endogenous GLP-1 secretion and circulating exposure. The specific novelty of the review lies in linking defined physicochemical changes to the identity, timing, intestinal location, and epithelial accessibility of candidate L-cell ligands across experimental models, while separating secretion from DPP-4 inhibition, direct GLP-1R agonism, and clinically meaningful outcomes. This framework is intended to improve the interpretation and design of functional-food studies.

## 2. Literature Search and Narrative Synthesis

This article was developed as a critical narrative review integrating evidence from food chemistry, gastrointestinal digestion, enteroendocrine biology, and human physiology. It was not intended to provide an exhaustive systematic review or quantitative meta-analysis. The formulation of the review question, description of the literature search, referencing, and presentation of the scientific argument were informed by SANRA guidance [21].

PubMed/MEDLINE was searched from database inception through 18 August 2026. To broaden coverage of the food-science and food-chemistry literature, Web of Science Core Collection, BIOSIS Citation Index, and Scopus were subsequently searched through 11 September 2026. The search combined GLP-1-related terms with terms describing food structure, processing, digestion, bioaccessibility, fermentation, and intestinal delivery:

(“GLP-1” OR “glucagon-like peptide-1” OR “L cell*” OR enteroendocrine) AND (“food matrix” OR “food structure” OR processing OR digestion OR bioaccessibility OR “cellular integrity” OR milling OR “particle size” OR viscosity OR gelation OR gelatinization OR retrogradation OR extrusion OR emulsion* OR interfacial OR encapsulat* OR “controlled release” OR “distal delivery” OR “protein aggregation” OR hydrolys* OR fermentation).

Named pharmacological GLP-1 receptor agonists were excluded. The search was applied to the topic field in Web of Science Core Collection and BIOSIS Citation Index and to the title, abstract, and keyword fields in Scopus. In Scopus, recent records were prioritized for the contemporary food-science update, while older foundational studies remained eligible through the other databases and reference-list screening.

Targeted searches were conducted to clarify specific nutrient-sensing pathways and processing mechanisms. The fixed PubMed/MEDLINE block (“GLP-1”[Title/Abstract] OR “glucagon-like peptide-1”[Title/Abstract] OR “L cell”[Title/Abstract] OR “L cells”[Title/Abstract] OR enteroendocrine[Title/Abstract]) was combined separately with each prespecified sensor or process term. Sensor terms included SGLT1, CaSR, PEPT1, FFAR1–4, GPR119, GPBAR1, TGR5, FXR, and TAS2R*, while process terms included particle size, cellular integrity, gelatinization, retrogradation, viscosity, emulsification, protein aggregation, hydrolysis, encapsulation, gastric emptying, intestinal delivery, and distal delivery. Reference lists of relevant primary studies and reviews were also examined for additional foundational or mechanistically informative publications.

Records were deduplicated using titles and DOIs and assessed iteratively according to their relevance to the review question. Eligible evidence included characterized foods, nutrients, digestion products, microbial metabolites, or molecular probes investigated in enteroendocrine cell models, primary tissues, organoids, isolated perfused intestine, animal models, or controlled human studies. Priority was given to studies that adequately described the tested exposure, comparator, dose, experimental model, and GLP-1 endpoint. Molecular docking alone, uncharacterized extracts, conference abstracts, and studies without a measured GLP-1 endpoint or a defined GLP-1-related mechanism were not considered sufficient evidence of physiological GLP-1 modulation.

Evidence was synthesized according to experimental model, intervention class, comparator, and measured endpoint. Cellular GLP-1 release was distinguished from circulating GLP-1 exposure, reduced degradation through DPP-4 inhibition, direct GLP-1 receptor agonism, and downstream outcomes such as glycaemia, appetite, and energy intake. Human studies were additionally classified according to whether they used a composition-matched comparator, water or placebo, or a structurally different food. The additional database searches broadened the evidence base, particularly for food-matrix design, cereal and starch processing, fermentation, controlled intestinal delivery, and protein extraction. However, they did not materially alter the central interpretation because processing-related differences in endogenous GLP-1 responses were not consistently accompanied by improvements in glycaemia, appetite, or energy intake.

## 3. Physiological and Analytical Basis for Interpreting GLP-1 Responses to Food

GLP-1 is produced by prohormone convertase 1/3 processing of proglucagon in intestinal enteroendocrine cells. The principal bioactive molecular forms are GLP-1 (7–36)NH_2_ and GLP-1 (7–37); dipeptidyl peptidase-4 (DPP-4) cleavage generates metabolites with little classical insulinotropic activity. Nutrient-responsive cells that express proglucagon occur throughout the intestine, although their density and hormonal coexpression vary along the proximal–distal axis. Enteroendocrine cells form a regional and transcriptionally heterogeneous continuum rather than a single immutable “L cell” phenotype. They may also secrete peptide YY, oxyntomodulin, neurotensin, or other hormones [1,2,3,22]. Proximal cells can contribute to the rapid postprandial GLP-1 rise even though tissue content is highest distally.

Several experimental layers have changed the definition of an L cell. Classical histology mapped cells that are immunoreactive for GLP-1 across the human gastrointestinal tract, with greater abundance distally. Dual labelling studies also showed that GLP-1 and GIP coexist in a subset of endocrine cells in the small intestine [23,24]. Receptor mapping in native mouse and human epithelia then demonstrated that multiple sensing systems coexist across regions and endocrine phenotypes [25]. Human organoid studies later showed plastic hormone identity along the BMP gradient from crypt to villus, established a detailed mRNA and secretome atlas, and resolved regional sensor repertoires at the level of individual cells [26,27,28]. Cell counts for one hormone cannot, on their own, predict secretion.

Regional heterogeneity also has functional consequences. Human duodenal and ileal L cells respond directly to glucose, predominantly through SGLT1, whereas the same ex vivo glucose stimulus did not elicit GLP-1 release from colonic tissue [29]. More recent single-cell profiling of human duodenal- and ileal-organoid enteroendocrine populations found SLC5A1 enriched in K and L cells, while the short-chain fatty-acid receptor FFAR2 and bile-acid receptor GPBAR1 were most prominent in ileal L-cell populations [28]. Colonic L cells are also exposed to microbial short-chain fatty acids and respond to these metabolites in experimental and targeted-delivery studies [30,31]. However, this regional specialization is not a simple proximal–distal dichotomy. In a randomized crossover trial involving 28 healthy adults, delivery of an identical short-chain-fatty-acid dose to the small intestine produced a greater GLP-1 incremental area under the curve, whereas colonic delivery produced a greater PYY incremental area under the curve [32]. The distinction is not absolute: in controlled human studies, ileal glucose delivery increased GLP-1 and PYY and reduced subsequent energy intake relative to duodenal delivery [33]; ileal protein reduced intake relative to duodenal delivery and increased GLP-1 relative to jejunal delivery and placebo [34]. Thus, the effect of food structure depends not only on ligand identity but also on the intestinal segment reached, the local sensor repertoire, and the timing of release.

Nutrients stimulate GLP-1 through electrogenic transport, metabolism, G-protein-coupled receptors and, in some cases, mechanosensory or neural pathways. The hormone acts locally on vagal and enteric afferents and, after entering the portal circulation, at endocrine targets. DPP-4 begins to inactivate GLP-1 rapidly, so intact hormone circulates for only a few minutes. Systemic concentration is a filtered representation of secretion, not a direct measure of mucosal release [1,2,3,35]. This brief physiology precludes assuming that an endogenous secretagogue reproduces the magnitude, duration, tissue exposure, or pharmacology of a degradation-resistant GLP-1 receptor agonist.

The rapid response to proximal nutrient entry need not require direct contact between every ligand and a distal L cell. Muscarinic signalling can stimulate GLP-1 secretion in a human enteroendocrine model, providing a basis for neural amplification [36]. In mice, intestinal GLP-1 also recruits an intestinofugal circuit linking the gut to gastric emptying and feeding behaviour [4]. A response after a meal may thus integrate chemosensing, epithelial communication, and neural feedback, while an experiment with a purified ligand isolates only part of that physiology.

Reports of a “GLP-1 effect” may refer to increased secretion, altered L cell differentiation or secretory capacity, reduced degradation by DPP-4, microbial generation of signalling metabolites, redistribution of ligand exposure by the food matrix, or direct GLP-1 receptor agonism. These mechanisms are not interchangeable. Inhibition of recombinant DPP-4 does not demonstrate L cell stimulation, and a rise in circulating GLP-1 does not demonstrate direct GLP-1R agonism. Direct agonism has not been established for an orally available, chemically defined food constituent in humans.

Measurement strategy depends on the proposed mechanism. Total GLP-1, measured with an assay that recognizes intact hormone and its principal DPP-4 metabolite, is generally the more informative circulating index of secretion. Intact or “active” GLP-1 reflects the combined effects of secretion, DPP-4 activity, sample handling, distribution, and clearance. When intact GLP-1 is an outcome, blood collection requires cooling and validated protease inhibition. Conversely, a rise in intact GLP-1 without a corresponding total GLP-1 response may indicate altered degradation rather than secretion. Recent mouse work illustrates how in vivo DPP-4 inhibition can reveal secretory responses that are otherwise obscured by rapid metabolism [37]. In human studies, reporting active and total GLP-1, assay specificity, sampling tubes, the time course and incremental area under the curve, and concomitant glucose, insulin, gastric emptying, and other gut hormone outcomes improves interpretation. Glucose lowering alone is not evidence of a GLP-1 mechanism.

## 4. Linking Food Chemistry with Intestinal L Cell Exposure

Foods often reach L cells as digestion products rather than as intact ingredients. Triacylglycerols yield non-esterified fatty acids and 2-monoacylglycerols; proteins yield free amino acids and oligopeptides; digestible carbohydrates yield monosaccharides; and fermentable carbohydrates generate microbial metabolites. Processing alters when and where these products are released and, consequently, which intestinal sensors they can reach. The relevant exposure is the composition of digesta at a particular intestinal site and time.

To distinguish these related terms, this review treats the food matrix as the complete physicochemical system, food structure as its multiscale organisation, and processing as the set of operations that modifies that organisation (Box 1).

Box 1 **Simple working definitions used in this review.**

Food matrix

   The food as a whole: its ingredients, water, phases, interfaces, and the interactions between them.

Food structure

   How the matrix is physically organised at three levels:

- Macroscopic: shape, particle size, texture, and viscosity.
- Microscopic: cells, cell walls, pores, gels, droplets, and interfaces.
- Molecular: the organisation and physical state of starch, proteins, lipids, and phytochemicals.

Processing

   Operations such as milling, heating, homogenisation, fermentation, and encapsulation that change the matrix or its structure.

Bioaccessibility

   The fraction released from the food during digestion and available for absorption or contact with an intestinal receptor.

GLP-1 relevance

   Processing → matrix or structure changes → altered digestion and nutrient release → altered timing and site of L-cell exposure → altered endogenous GLP-1 response.

In this review, bioaccessibility refers to the fraction of a constituent or digestion-derived product released from the food matrix into gastrointestinal fluids and available for absorption or luminal receptor contact. It is distinct from bioavailability, which additionally requires epithelial uptake and delivery to the circulation or target tissue, and from biological activity, which requires a measurable molecular or physiological effect. For GLP-1, this distinction is critical: a compound may be released during digestion yet fail to reach the relevant apical or basolateral sensor, whereas an accessible ligand may be inactive at the concentration achieved. Standardised in vitro digestion models can estimate release and transformation but cannot establish epithelial transport, L-cell secretion, or a human postprandial response [38,39].

Accordingly, Section 5 asks which chemical species and sensors can generate a GLP-1-related signal, whereas Section 6 asks how processing and structure determine whether, when, and where those species become bioaccessible.

The physiological relevance of an experiment depends on whether that exposure is plausible. Receptor docking of a peptide that is never liberated during digestion, for example, has little bearing on food intake. Adding a poorly absorbed polyphenol aglycone to the basolateral surface of an unpolarised cell line also misrepresents its predominantly glycosylated luminal exposure after a meal. In human enteroendocrine organoids, disruption of receptors using CRISPR supports roles for CaSR, FFAR2, FFAR4, GPR119, and GPBAR1/TGR5 under defined conditions [40]. Overnight micromolar or millimolar exposure nevertheless shows cellular capacity rather than the concentration, polarity, or time course produced by an ordinary serving.

Processing changes both ligand identity and delivery kinetics. Heating and gelatinization generally accelerate starch accessibility, whereas retrogradation and intact cellular structures can shift carbohydrate toward distal digestion or fermentation. Processing effects are not uniform. Extrusion increased starch damage and lowered resistant starch in formulations containing corn and spelt. By contrast, extrusion or roasting in the presence of wastewater from apple distillation that was rich in malate generated starch esters with greater resistance to amyloglucosidase [41,42]. Protein denaturation may facilitate proteolysis, but aggregation, crosslinking, or advanced Maillard chemistry may reduce enzyme accessibility and alter the peptide repertoire.

Fermentation and enzymatic hydrolysis can generate small peptides, free amino acids, organic acids, or microbial metabolites before consumption. Hydrolysis followed by fractionation with a 3 kDa membrane shifted the peptide size distribution of proteins from fish processing side streams. Whey fermentation produced different time courses of free amino groups and peptides of low molecular weight in monoculture and coculture [43,44]. Neither study measured GLP-1. They characterise the potential ligand pool rather than endocrine efficacy. Emulsification increases the interfacial area available to lipase and may accelerate the release of fatty acids and 2-monoacylglycerols. Acyl chain length, unsaturation, and stereospecific position on glycerol determine both lipid physical properties and the ligands produced during digestion. Polyphenol glycosylation, oxidation, polymerization, and binding to protein or polysaccharide govern whether the parent compound, a conjugate, or a colonic catabolite reaches the epithelium.

Regional delivery and epithelial polarity are equally important. Responses involving CaSR or TGR5 can be stronger from the basolateral compartment in intact tissue, whereas SGLT1 and PEPT1 are primarily apical transport systems [29,45,46,47]. Some ligands must therefore undergo absorption, transport across the epithelium, bile acid recycling, or lipoprotein processing before reaching the relevant sensor. Unpolarised immortalised cell lines cannot reproduce this topology. Molecular docking, cell-free inhibition, and immortalised-cell assays identify candidate interactions. Polarised primary cells or organoids, receptor perturbation, perfused intestine, animal interventions, and controlled human studies address different parts of the mechanism.

Complex meal responses cannot usually be assigned to one receptor. A mixed meal containing protein, lipid, and carbohydrate can simultaneously engage transporters, GPCRs, bile acid signalling, gastric distension, neural reflexes, and delayed microbial pathways. Food may also raise GLP-1 indirectly by slowing gastric emptying or changing nutrient delivery. Receptor antagonists, gene deletion, targeted intestinal delivery, equimolar ligand comparisons, and measurement of compounds in digesta can help distinguish association from causality. Interpretation therefore depends on the chemical identity, concentration, location, and timing of the ligand that reaches the sensor. Figure 1 summarises representative routes by which digestion products engage polarised nutrient-sensing pathways and highlights the distinctions among endogenous GLP-1 secretion, DPP-4 inhibition, direct GLP-1 receptor agonism, and downstream physiological outcomes.

## 5. Chemical Routes from Food to Endogenous GLP-1 Secretion

### 5.1. Carbohydrates, Rare Sugars, and Sweeteners

Glucose provides the clearest example of GLP-1 secretion through transporter coupling. Apical uptake through sodium–glucose cotransporter 1 (SGLT1) is electrogenic: cotransport of Na^+^ depolarizes the enteroendocrine membrane, opens voltage-gated Ca^2+^ channels, and initiates exocytosis. Human intestinal experiments and genetic or pharmacological rodent studies place SGLT1 upstream of a substantial component of GLP-1 release induced by glucose [29,48,49]. Intracellular glucose metabolism and basolateral glucose handling may amplify the response, but the evidence does not justify treating sweetness itself as the primary signal.

The intestinal T1R2/T1R3 sweet taste receptor and its signalling partners are expressed in enteroendocrine tissue, and some pharmacological studies support a contribution to glucose sensing [50]. Experiments in perfused intestine and inconsistent hormonal effects of non-nutritive sweeteners indicate that this pathway is neither necessary nor sufficient for the normal acute response [51]. A 2023 systematic review and network meta-analysis found no consistent acute GLP-1 increase after beverages sweetened with non-nutritive compounds compared with water [52]. “Sweetener” is a sensory rather than mechanistic category. Structurally unrelated sweeteners differ in receptor pharmacology, absorption, osmotic activity, microbiome exposure, and dose, so their effects should not be pooled as one chemical class.

The rare sugar D-allulose and the polyol erythritol illustrate this distinction. In a small randomised human study, both increased postprandial GLP-1, yet lactisole did not abolish the effect, arguing against a simple T1R2/T1R3 mechanism [53]. Rat work then implicated luminal distension in secretion induced by D-allulose, suggesting contributions from poor absorption and osmotic water retention [54]. A 2026 mouse study showed that endogenous GLP-1 evoked by D-allulose engages vagal signalling dependent on GLP-1R and insulin [55]. D-Allulose behaved as a secretagogue under the tested conditions; it was not a direct GLP-1R agonist. Human dose tolerance, gastrointestinal symptoms, habitual exposure, and the contribution of distension require resolution before this mechanism informs food design.

For foods rich in starch, the relevant variables are the rate and site of glucose appearance and the fraction escaping digestion in the small intestine. Rapidly digestible starch may generate a stronger proximal SGLT1 signal but also a larger glycaemic load. Resistant or structurally encapsulated starch can reduce this proximal stimulus while increasing distal fermentation. A lower early GLP-1 response need not imply a lower integrated response; the observation window must distinguish monosaccharide sensing over minutes from microbial signalling over hours.

### 5.2. Proteins, Amino Acids, and Peptides from Food

Protein is often a robust stimulus in a whole meal, but “protein” is chemically too coarse to explain receptor activation. Gastric and pancreatic proteolysis generates a dynamic mixture of free amino acids and oligopeptides whose sequence, charge, hydrophobicity, size, and digestive stability depend on the source protein and processing history. Proton-coupled uptake of dipeptides and tripeptides through PEPT1 can depolarize enteroendocrine cells, while peptones and selected amino acids can activate the calcium-sensing receptor (CaSR). In intact perfused intestine, GLP-1 release induced by peptones requires absorption and is compatible with predominantly basolateral CaSR sensing [45,46]. Human organoid receptor knockout now provides direct evidence from human cells that CaSR senses amino acids [40].

Individual amino acids do not behave uniformly. Glutamine increases intracellular Ca^2+^ and cAMP in enteroendocrine cells and, at pharmacological oral doses, raises circulating GLP-1 in lean, obese, and type 2 diabetic participants [56,57]. Oral L-arginine also increased postprandial GLP-1 and PYY in a human intervention [58]. In contrast, the candidate orphan receptor GPRC6A was not required for robust secretion induced by basic amino acids in mice [59]. Systematic comparisons in perfused intestine show marked responses specific to each amino acid and intestinal region, with transporter coupling, metabolism, CaSR, and other pathways contributing to different extents [60]. L-Valine was particularly potent in rodents, apparently through intracellular metabolism, closure of ATP-sensitive potassium channels, depolarization, and Ca^2+^ entry rather than a defined extracellular GPCR; human confirmation is still absent [61].

Whey preloads increase circulating GLP-1 and improve postprandial glycaemia in type 2 diabetes [62,63]. The human studies establish an effect at the level of a meal but do not identify a single whey peptide or receptor. Whey is rapidly digested, produces high exposure to amino acids, stimulates insulin and several gut hormones, and can slow gastric emptying. These findings support secretion in response to products of whey digestion, not an effect of intact whey as a GLP-1 mimetic [9].

Studies of peptides with known sequences provide a firmer chemical basis than crude hydrolysates. A pea protein hydrolysate stimulated GLP-1 in NCI-H716 cells through mechanisms involving CaSR and PEPT1 [64]. Screening guided by CaSR later identified the pea peptides RFY, FEPF, and FLFK, with FEPF and FLFK showing relative digestive stability [65]. The sequence WIRGCRL, derived from lysozyme, stimulated GLP-1 in mouse jejunal organoids and raised plasma GLP-1 during a glucose challenge in rats. Alanine substitution modified activity, supporting a sequence–activity relationship [66]. Peptides recovered from the human jejunum after milk digestion have also induced enteroendocrine hormone secretion in vitro [67]. None of these experiments yet demonstrates a reproducible human GLP-1 effect after administration of a defined peptide.

Peptides from food that inhibit DPP-4 require a separate interpretation. Numerous short peptides inhibit purified DPP-4, often with hydrophobic or proline-containing N-terminal motifs [68,69]. Potency in cell-free assays, however, may be lost through brush border proteolysis, mucus binding, low epithelial permeability, dilution, and metabolism. Caco-2 studies show that only a fraction of candidate milk peptides crosses the intestinal barrier intact [68]. A peptide can theoretically be both a secretagogue for L cells and a DPP-4 inhibitor, but these activities require separate assays and exposure estimates. Increased intact GLP-1 after a hydrolysate is insufficient to assign either mechanism unless total GLP-1 and DPP-4 activity are measured concurrently. None of these peptide findings demonstrates direct GLP-1R agonism.

### 5.3. Dietary Lipids and Lipolysis Products

Triacylglycerol is mainly a precursor rather than the immediate ligand for L cells. Gastric and pancreatic lipases release non-esterified fatty acids and predominantly sn-2 monoacylglycerols, which can engage several sensors. Long-chain fatty acids activate FFAR1/GPR40 and FFAR4/GPR120, generally through Gq-linked Ca^2+^ signalling, although the relative contribution varies with species, intestinal region, ligand, and model [70]. Chain length matters: intraduodenal lauric acid elicited stronger GLP-1 and related gastrointestinal effects than decanoic acid in humans [71]. Degree and geometry of unsaturation may also alter potency, but many structure–activity claims still rely on cell assays at high concentrations rather than equimolar intestinal exposure.

The strongest chemically resolved human lipid pathway involves GPR119. The digestion product 2-oleoylglycerol acts as a GPR119 agonist and stimulates GLP-1 release in humans [72]. A subsequent intervention supported the 2-monoacylglycerol moiety, rather than fatty acid alone, as an important mediator of GLP-1 secretion induced by fat [73]. Oleoylethanolamide also stimulates GLP-1 through GPR119 in preclinical models [74]. For food design, the key variable is the 2-monoacylglycerol generated during lipolysis, which depends on acyl identity and stereospecific position. Interesterification, emulsification, droplet size, and modulation of lipase can change ligand generation even when total fat and fatty acid composition are nominally identical.

An additional route may arise after enterocyte uptake. Chylomicrons stimulate incretin secretion in mouse and human enteroendocrine models, possibly after fatty acids are liberated by lipoprotein lipase at the basolateral surface [75]. Basolateral processing may explain why responses in intact tissue depend on membrane side and why a purely luminal assay can miss a relevant lipid signal. The evidence supports dietary fat digestion products as secretagogues acting through FFARs, GPR119, transport, and postabsorptive lipid processing. It does not support describing dietary lipids as direct GLP-1R agonists.

### 5.4. Bile Acids as Signals Linked to Meals and Modified by Microbes

Bile acids occupy an intermediate position between host digestion chemistry and microbial metabolism. A meal containing fat induces gallbladder emptying, whereas intestinal bacteria subsequently deconjugate and transform primary bile acids into structurally distinct secondary species. Bile acids stimulate GLP-1 through the membrane receptor GPBAR1/TGR5, with hydrophobic secondary bile acids such as lithocholic and deoxycholic acids generally more potent than primary bile acids. In perfused intestine, the response is markedly stronger from the basolateral side, indicating that epithelial transport and enterohepatic cycling determine access to TGR5 [47].

Bile acid signalling is not uniformly stimulatory. The nuclear receptor FXR can suppress proglucagon expression and GLP-1 production in L cells [76]. Total bile acid concentration alone cannot predict the meal response; conjugation, hydroxylation pattern, microbial conversion, transporter access, and the balance between TGR5 and FXR all matter. A food-related bile acid mechanism is therefore easier to interpret when targeted profiling shows that altered molecular species reach the relevant intestinal compartment. This is an indirect secretory route linked to meals, not direct GLP-1R agonism.

### 5.5. Phytochemicals and Bitter Taste Ligands

Intestinal bitter taste receptors of the TAS2R family provide an attractive link between phytochemical structure and enteroendocrine function, but the receptor family is large, differs among species, and is pharmacologically promiscuous. The plant alkaloid berberine stimulated GLP-1 release in a human enteroendocrine cell line through a mechanism involving TAS2R38 [77]. Quinine has provided human proof of principle: intraduodenal administration alters gut hormone release, with effects depending on dose, delivery site, and sex [78]. In men with type 2 diabetes, 600 mg produced a modest GLP-1 response, whereas the lower dose was less effective [79]. Such doses are closer to pharmacological probes than to normal culinary exposure and may be limited by bitterness and gastrointestinal tolerability.

Evidence for individual receptor subtypes remains scarce. The synthetic bitter probe 1,10-phenanthroline stimulated secretion in a human enteroendocrine line through TAS2R5 signalling [80]. The experiment identifies the ligand, receptor subtype, signalling response, and secretion, but 1,10-phenanthroline is not a food exposure and cannot validate a botanical mixture.

Polyphenols are frequently proposed as agents that enhance GLP-1, but attribution is difficult. Glycosides, aglycones, phase II conjugates, oxidation products, and microbial catabolites differ in receptor access and may affect carbohydrate digestion, transporters, DPP-4, bile acids, inflammation, or the microbiome in parallel [81]. A randomised trial combining catechins and chlorogenic acids altered glucose and incretin outcomes in healthy men, but the mixture design could not identify a ligand and sensor pair [82]. An increase after an extract rich in polyphenols is therefore an association with the mixture unless receptor disruption and measurement of the bioavailable active structure support a narrower claim. Molecular docking to TAS2R, DPP-4, or GLP-1R can only generate a hypothesis. No phytochemical in this evidence set is an established direct GLP-1R agonist at a dose achievable from food in humans.

### 5.6. Microbial Metabolites

Fermentable carbohydrates and other substrates may influence L cells after microbial conversion into signalling metabolites, including short-chain fatty acids and secondary bile acids [83]. Acetate, propionate, and butyrate stimulate GLP-1 in experimental systems, with the strongest receptor evidence generally favouring FFAR2/GPR43 [84,85]. FFAR3/GPR41 involvement is less consistent, and short-chain fatty acids can also act through metabolism, histone deacetylase inhibition, neural pathways, and changes in epithelial energy status. Experiments in perfused colon show that individual short-chain fatty acids differ in potency and can produce effects not fully predicted by receptor expression [30].

A standardized carob formulation subjected to INFOGEST digestion and faecal fermentation showed phase-dependent activity in STC-1 cells: GLP-1 release was strongest after gastric digestion, more modest after intestinal digestion, and transient during early fermentation [86]. This finding extends food-science coverage but remains an in vitro result; it supports treating the digest or fermentate, rather than the starting ingredient alone, as the relevant exposure.

One programme of targeted delivery found that colonic propionate increased postprandial GLP-1 and PYY and, during longer administration, reduced energy intake and weight gain [31]. This provides stronger proof of concept than correlations between faecal short-chain fatty acids and plasma GLP-1, but it is not an independent replication across programmes. A chemically modified inulin–propionate ester is also not equivalent to ordinary fibre. For intact foods, polymer linkage, solubility, particle structure, fermentability, microbial ecology, production rate, absorption, and colonic location determine exposure. Faecal concentration can even vary inversely with epithelial uptake and is an unreliable surrogate for exposure at the receptor.

Indole produced from tryptophan illustrates why exposure time matters. In L cells, acute indole exposure can increase GLP-1 secretion through effects on membrane excitability, whereas prolonged exposure suppresses secretion by reducing mitochondrial metabolism [87]. Secondary bile acids provide another class of ligands generated through microbial activity, as discussed above. Reviews that group short-chain fatty acids, bile acids, and indoles as signalling between the microbiome and GLP-1 risk obscuring their opposing actions and dependence on molecular structure [88].

The clearest structure–activity relationships involve molecules demonstrably generated during digestion or fermentation. They include glucose acting through SGLT1, selected amino acids and oligopeptides acting through transport and CaSR pathways, long-chain fatty acids acting through FFAR1/4, and 2-monoacylglycerols acting through GPR119. Molecularly defined bile acids act through TGR5 and FXR, while short-chain fatty acids can act through FFAR2. Human GLP-1 responses have been demonstrated for SGLT1-mediated glucose sensing, GPR119 activation by 2-oleoylglycerol, whey-containing meals, targeted propionate delivery, and pharmacological quinine. Evidence for defined food peptides, individual phytochemicals, and direct GLP-1R agonism remains limited. Whether these candidate ligands reach polarised human intestinal tissue at concentrations produced by ordinary foods often remains unknown.

## 6. Processing and Food Structure: Effects on Nutrient Sensing

### 6.1. Cellular Integrity, Milling, and Particle Architecture

Milling changes more than particle size. It ruptures plant cell walls, increases the surface area accessible to enzymes, damages starch, releases intracellular protein and lipid, and alters hydration. The clearest human proof of principle comes from chickpea and includes one randomised crossover pilot with interventions matched for composition and direct gastric and duodenal sampling. Broken cells generated rapid gastric maltose accumulation and early GLP-1 release, whereas intact cells remained visible in the upper gut and produced later, more sustained GLP-1 and PYY responses [17]. Two bread studies extend the feasibility evidence but are not independent replications with matched composition. Cellular chickpea flour sustained GLP-1 and PYY secretion [89], while chickpea flour with larger particles was associated with lower glycaemia and higher GLP-1 than refined wheat bread despite compositional differences between the breads [90]. Oat flakes and flour also differed in glycaemia and early gut hormone kinetics despite comparable initial gastric emptying [91]. Pyloric sieving and starch accessibility may therefore matter more than the overall gastric emptying rate. The label “coarse” is insufficient. Useful structural descriptors include cell wall continuity, particle size distribution, porosity, damaged starch, and persistence during digestion.

Food engineering provides a complementary human example. Microfluidic extrusion and gelation were used to create calcium-alginate/soy-protein noodle-like assemblies with tunable internal spatial segregation and texture. The assemblies produced different GLP-1 and ghrelin profiles from commercial noodles and from one another, while postprandial glucose, insulin, and subjective satiety changed little [92]. This result illustrates endocrine differences after food engineering but also shows that a hormone difference need not translate into perceived satiety.

### 6.2. Viscosity, Gelation, and Starch State

Viscosity can slow convective transport, enzyme diffusion, and gastric emptying, but it does not predict the direction of the GLP-1 response. In isoenergetic oat bran beverages, enzymatically lowering viscosity accelerated gastric emptying and produced larger early GLP-1, PYY, and CCK responses [93]. Increasing oat β-glucan viscosity, by contrast, delayed emptying and reduced glycaemia without improving PYY, appetite, or food intake [18]. Oat bread enriched with β-glucan also delayed emptying and lowered glucose and insulin while leaving GLP-1 unchanged [19]. The direction depends on when and where nutrient delivery is measured. A viscous matrix may flatten proximal ligand delivery enough to reduce an early hormone peak without increasing distal exposure within the observation window.

Thermal history provides another control point. Gelatinization generally increases amylase accessibility, whereas limited gelatinization, amylose association, and retrogradation can delay glucose release. A lower glycaemic response, however, is not evidence of greater GLP-1 secretion: a resistant-starch/pullulan combination acutely lowered both glucose and GLP-1 [94], and high-amylose resistant starch failed to raise GLP-1 acutely [95]. Reporting rapidly available, slowly available, and resistant starch fractions after preparation as consumed avoids inferring hormonal effects from ingredient specifications.

A recent synthesis argues that starch functionality should be described across molecular, granular, and matrix scales because each can alter the rate and location of digestion [14]. In mice, competitive α-amylase inhibition by luteolin or quercetin extended complete starch digestion distally, increased GLP-1, and reduced food intake and weight gain [96]. The latter is preclinical and does not establish that flavonoid-containing foods will reproduce the effect in humans.

Thermal processing can act in the opposite direction. In mice, boiling, baking, or frying rice starch reduced branching density and structural order, accelerated digestion, increased postprandial glucose and insulin, and lowered GLP-1 relative to raw starch [97]. Although direct translation to prepared foods in humans is uncertain, the study links a defined structural change to digestive kinetics and the endocrine response.

### 6.3. Lipid Emulsions and Interfacial Chemistry

For lipids, total fat and fatty acid composition are incomplete descriptors. Droplet size, interfacial composition, gastric acid stability, coalescence, creaming, and lipid crystallinity determine lipase access and the delivery of free fatty acids and monoacylglycerols to intestinal sensors. Emulsions that remain stable under gastric acid conditions can delay gastric emptying relative to emulsions that separate in the stomach [98]. With much of the composition held constant, liquid rather than partially crystalline droplets strengthened acute satiety signals [99]. In a factorial human study, the liquid-lipid, acid-stable emulsion delayed gastric emptying and increased the 6 h incremental area under the plasma GLP-1 concentration–time curve by 38% versus the solid-lipid acid-unstable formulation and by 76% versus the liquid-lipid acid-unstable formulation [20]. Interfacial area and the lipolysis trajectory after oral and gastric processing are more informative than droplet size measured only in the manufactured product.

### 6.4. Protein Denaturation, Aggregation, and Hydrolysis

Thermal unfolding, aggregation, gelation, mineral bridging, and enzymatic hydrolysis alter solubility, gastric structuring, protease access, peptide size distribution, and sensory properties. The response depends on the substrate. Calcium increased GLP-1 only when ingested with aggregated β-lactoglobulin, whereas its effect on gastric emptying occurred with the native form [100]. Earlier intragastric work found similar GLP-1 and PYY responses to intact casein, intact whey, and their hydrolysates despite differences in gastric secretion and GIP [101]. Digests of casein and whey can stimulate GLP-1 release in cell models, but the active digestion phase differs by substrate [102]. A casein hydrolysate active in cells and mice still constitutes preclinical evidence [103]. In humans with type 2 diabetes, intact and hydrolysed whey produced similar incretin responses [104]. Pea hydrolysates that differed in bitterness and degree of hydrolysis changed early proxies for gastric emptying, plasma DPP-4 concentration, ghrelin, and intake without changing GLP-1 [105]. Degree of hydrolysis is a process descriptor, not a biological mechanism. Peptidomics after simulated and, where feasible, human digestion could help identify the ligands actually presented to sensors.

Two food-chemistry studies sharpen the distinction between the manufactured material and intestinal exposure. Micellar casein concentrate and a sodium-caseinate/whey-protein blend matched the casein:whey ratio generated by different peptide patterns during simulated gastrointestinal digestion and different GLP-1 release in GLUTag cells; the peptide GPVRGPFPIIV was identified as active [106]. By contrast, water-soluble extracts of ripened Irish Cheddar stimulated GLP-1 in STC-1 cells, but the most potent extract lost this activity during the intestinal phase of simulated digestion [107]. These results reinforce that bioactivity measured in an undigested extract cannot be assumed to survive digestion.

In soy-protein systems, cultivar and extraction severity altered protein solubilisation during simulated digestion, D-amino-acid formation, DPP-4 inhibition, and total GLP-1 release in vitro [108]. The most severe alkaline extraction increased protein yield and DPP-4 inhibition but reduced post-digestion solubilisation and increased D-amino-acid formation, showing that extraction yield and intestinal bioactivity need not improve together.

### 6.5. Controlled Release and Distal Nutrient Delivery

Encapsulation can shift sensing distally, but interpretation depends on establishing the release location. Direct ileal protein infusion reduced subsequent intake relative to duodenal infusion and placebo, whereas GLP-1 increased relative to jejunal infusion and placebo [34]. Duodenal and ileal glucose delivery also produced different peptide and appetite profiles [33]. These experiments establish positional causality, not the efficacy of an oral food. One programme of targeted delivery showed that inulin–propionate ester chemistry controls colonic release and acute intake effects [109]. The related longer intervention is discussed in the section on human evidence [31]. Results are less predictable for other payloads. Glutamine formulated for ileal release produced only a small, inconsistent GLP-1 signal and no useful metabolic effect [110]. An encapsulated casein–sucrose mixture reduced intake, but gut hormones were not measured [111]. Delivery of α-linolenic acid via porous silicon prolonged GLP-1 secretion in mice and remains a preclinical proof of concept [112]. Endocrine interpretation also depends on the measured payload, encapsulation efficiency, sequential release kinetics, and intestinal recovery.

In a small randomized crossover study of 10 participants after Roux-en-Y gastric bypass and 10 matched unoperated participants, slowly digested isomaltulose increased the 4 h positive incremental area under the total GLP-1 curve by 170% versus sucrose in the unoperated group; the 50% increase after surgery was not statistically significant [113]. A separate proof-of-concept study engineered a resistant-starch–lecithin nanoparticle carrying β-lactoglobulin; colonic fermentation released the payload and short-chain fatty acids and increased GLP-1 in NCI-H716 cells relative to comparator formulations [114]. Together, these studies support digestion rate, anatomy, and release location as determinants of sensing, but the small, selected human sample and in vitro delivery model limit generalization.

Table 1A,B summarise chemical ligands and signalling mechanisms, whereas Table 2 summarises the processing operations and structural changes that govern their release and intestinal delivery.

### 6.6. Processing Intensity and Additives: Avoid Inference from Processing Category Alone

“Ultra-processed” is not a physicochemical mechanism. An inpatient trial showing greater energy intake on an ultra-processed diet establishes an effect of the dietary pattern [118], but does not identify GLP-1 or any individual processing operation as the mediator. A cell study found that λ-carrageenan suppressed proglucagon expression and GLP-1 secretion [119], but this does not show that carrageenan lowers GLP-1 in consumers. For thermal reactions, oxidation, emulsifier exchange, and interactions between additives and the matrix, interpretation depends on the chemical species formed, their dose and bioaccessibility, and the measured endocrine response rather than the processing label [120].

The NOVA classification is useful for population-level description, but its categories should not be treated as physicochemical exposures. Processing-based systems combine distinct dimensions—including the extent and nature of modification, formulation, place, and purpose of processing—and generally lack quantitative thresholds [121]. Consistent classification is also difficult: agreement among food and nutrition specialists was low even when ingredient information was supplied, and products assigned to NOVA 4 spanned markedly different nutritional profiles [122]. Mechanistic interpretation should therefore identify the specific unit operations, formulation changes, resulting structures, and chemical species rather than attribute an endocrine effect to the ‘ultra-processed’ label itself.

## 7. Human Evidence: Separating GLP-1 Secretion from Clinically Meaningful Efficacy

### 7.1. The Gap Between Secretion and Efficacy

Meal composition, food structure, and the kinetics of nutrient delivery can modify circulating GLP-1. No human study, however, justifies describing a food as the physiological equivalent of a long-acting GLP-1 receptor agonist. Native GLP-1 is secreted in short postprandial pulses and is rapidly cleaved by dipeptidyl peptidase-4 (DPP-4). Its intact circulating half-life is approximately 1–2 min [11,12,13]. Even a large relative increase from a low picomolar baseline does not establish sustained receptor exposure, appetite suppression, or weight loss. Its physiological relevance therefore depends on whether a tolerable, nutritionally realistic intervention changes an independent endpoint and whether GLP-1 contributes causally.

Controlled infusion studies help define this boundary because they hold exposure more steadily than a meal can. At physiological concentrations in men with obesity, GLP-1 markedly slowed gastric emptying and reduced hunger but did not reduce ad libitum energy intake [123]. Another small study prolonged postprandial satiety without changing meal size [124]. By contrast, an infusion lasting eight hours reduced intake across two meals in eight men with severe obesity [125]. Experiments across several doses showed that inhibition of gastric emptying can outweigh insulinotropic effects [126]. Repeated exposure within the same day also produced rapid tachyphylaxis of the emptying response [127]. Exposure duration, concentration, prior stimulation, gastric motility, and choice of endpoint all influence whether a hormone signal changes eating behaviour. A transient dietary increment warrants more conservative interpretation than a sustained infusion.

Trials using mixed meals show a similar disconnect. A plant meal matched to the comparator for energy and macronutrients increased GLP-1 and subjective satiety compared with a meal based on processed meat and cheese in men with type 2 diabetes, obesity, or normal weight [128]. In 12 people with type 2 diabetes, a Mediterranean meal produced higher GLP-1 and oxyntomodulin and lower postprandial glucose than a vegetarian meal high in fibre, but appetite ratings did not differ [129]. After two years of weight loss induced by diet, postprandial GLP-1 increased in both the Palaeolithic and control diet groups. Weight loss or metabolic adaptation may therefore dominate over a unique dietary pattern [130]. Meal sequence can improve glycaemia while increasing GLP-1, but fish or meat consumed before rice markedly delayed gastric emptying [131]. GLP-1 was one component of a coupled physiological response, not an isolated mechanism. Evidence from whole diets and meal patterns cannot support attribution to a single bioactive compound without stronger mechanistic controls.

### 7.2. Human Findings by Intervention Class

Among protein-based interventions, whey protein has the most reproducible acute human evidence. Protein preloads increase GLP-1, GIP, and insulin, slow gastric emptying, and lower postprandial glucose, particularly in type 2 diabetes [63,132]. These responses vary with dose and context and cannot be assigned exclusively to GLP-1. Among 79 participants with type 2 diabetes who completed a 12-week trial, 37 received a whey/guar preload and 42 received a flavoured, negligible-energy control. HbA1c was about 0.1 percentage point lower with whey/guar, without detectable differences in body weight or energy intake; postprandial total GLP-1 concentrations were lower at weeks 1 and 12 despite improved glycaemia [133]. A crossover trial of a premeal whey protein microgel reported a 22% reduction in glucose iAUC and a 66% increase in total GLP-1 iAUC, both over 0–2 h [134]. Insulin also increased, and early acetaminophen appearance was delayed, although the gastric emptying proxy over 4 h did not differ overall. The water comparator, added preload energy, commercial nature of the product, and manufacturer support limit inference about the mechanism. In a separate 2024 trial, aggregated versus native β-lactoglobulin and calcium produced different responses in total GLP-1 and gastric emptying, yet none clearly reduced subsequent energy intake [100]. Whey preloads therefore have more consistent support for acute postprandial glycaemic modulation than for GLP-1-mediated weight control.

Macronutrient effects are not simply additive. In a controlled crossover study, substituting carbohydrate and fat for part of a whey load changed gastric emptying, ghrelin suppression, and GLP-1. Adding the same macronutrients to a larger protein load did not reproduce those changes [135]. The complete formulation and its delivery kinetics are the appropriate experimental unit.

Human findings for fibres and metabolites generated by fermentation vary because fibre materials differ in viscosity, fermentability, molecular weight, and retention within the food matrix. One programme targeting propionate delivery to the colon found that inulin–propionate ester reduced acute energy intake and attenuated weight gain over 24 weeks [31]. Acute increases in circulating short-chain fatty acids induced by inulin, however, did not increase GLP-1 or PYY [136]. A resistant-starch/pullulan combination lowered glucose and insulin while reducing GLP-1 and leaving satiety unchanged [94]. Oat bread enriched with β-glucan improved glycaemia and delayed gastric emptying without increasing GLP-1 [19]. In two related chickpea studies from an overlapping research programme, cellular flour prolonged gut hormone profiles [89], while direct gastric and duodenal sampling in an experiment with conditions matched for composition supported delayed nutrient release [17]. The second study strengthens the mechanism but is not a fully independent replication. A longer oligofructose trial also found poor alignment between GLP-1 and appetite outcomes across fibre comparators [137]. A fibre can therefore improve metabolism without GLP-1 mediation. Because colonic fermentation occurs after conventional tests lasting 2–3 h, studies of this pathway are more informative when they include repeated exposure, later sampling, and measurement of circulating rather than faecal short-chain fatty acids.

Findings from two human studies further supported this discordance. In a 44-participant blinded crossover trial, wholemeal breads produced with yeast, sourdough, or both generated modest differences in GLP-1, PYY, C-peptide, eating rate, and satiety, but not in gastric emptying, glucose, or subsequent energy intake [138]. In 20 participants, beverages containing intact, hydrolysed, or extruded oat bran similarly reduced postprandial glucose and insulin; intact and extruded products produced lower, rather than higher, 3 h GLP-1 exposure than the control [139]. Together, these findings strengthen the evidence that processing-dependent GLP-1 changes can be dissociated from glycaemic, appetite, and intake outcomes.

Among lipid-derived ligands, long-chain fatty acids can stimulate GLP-1 and slow gastric emptying, but the response is inseparable from energy load and gastrointestinal tolerability. Intraduodenal lauric acid increased GLP-1 and reduced subsequent intake in a small human experiment, but caused nausea at the effective exposure [71]. Saturated and monounsaturated fats differ in their postprandial incretin and lipoprotein responses [140], and extra virgin olive oil has altered gastric emptying and glycaemia in small studies [141]. The studies support sensing of fatty acids, but not routine addition of lipid as a strategy against obesity, particularly because lipid is energy dense.

Human evidence for polyphenols and bitter compounds is limited to small, acute studies that generally lack independent replication. Cinnamon increased GLP-1 in healthy participants without improving glucose, satiety, or gastric emptying [142]. Oleuropein improved postprandial glucose handling and incretin responses in a crossover study [143]. In people with type 2 diabetes, 600 mg intraduodenal quinine modestly increased postdrink GLP-1 versus water, whereas 300 mg did not; the two doses did not differ significantly. Quinine also delayed gastric emptying and lowered early glycaemia without reducing energy intake [79]. In another experiment, intraduodenal delivery of a tastant mixture reduced food intake despite no change in GLP-1, PYY, or CCK [144]. Delivery route and dose are decisive. Direct gastric or duodenal dosing does not show that an ordinary bitter food will reproduce the effect.

Evidence for fermented foods and probiotics depends on the strain, matrix, and population. Four weeks of *Lactobacillus reuteri* increased GLP-1 and insulin secretion after oral glucose in adults with normal glucose tolerance but did not improve insulin sensitivity or body composition [145]. A trial lasting 10 weeks found that yogurt fortified with probiotics or vitamin D was associated with higher fasting GLP-1 during energy restriction [146]. Concurrent treatment for weight loss, baseline adjustment, and reliance on fasting hormone concentrations limit causal attribution. Neither study identified a chemical mediator linking the intervention to L cell exposure, so the findings cannot be generalised from these products to fermented foods as a class.

Across these intervention classes, heterogeneity is expected from systematic differences in dose and energy load, ligand chemistry, physicochemical form and matrix retention, site and route of delivery, sampling duration, comparator design, and participant phenotype. A water comparator estimates the net effect of consuming an energy-containing product but cannot isolate food structure; a composition-matched or process-matched comparator provides stronger structural attribution. Short tests may miss colonic fermentation, whereas longer adaptation may change microbial metabolism. Gastric emptying can shift both the GLP-1 curve and glycaemia, and nausea can lower intake independently of GLP-1. Finally, fasting concentration, peak concentration, total AUC, and incremental AUC are not interchangeable outcomes. These design differences explain why an intervention may alter GLP-1 in one study but not another and why hormone, appetite, glycaemic, and intake outcomes are frequently discordant (Table 3).

### 7.3. Causality and Clinical Meaning

Correlations between GLP-1 AUC and appetite ratings do not establish mediation. In a modelling analysis of data from controlled low energy diets, circulating GLP-1 and PYY did not consistently predict increased satiety [147]. GLP-1 receptor blockade with exendin(9–39) establishes that endogenous GLP-1 contributes to postprandial glucose regulation [148,149]. Its acute effects on appetite and ad libitum intake, however, are weak or inconsistent [150,151]. A rise in GLP-1 induced by food is neither necessary nor sufficient for reduced energy intake.

Population characteristics can alter both baseline and stimulus-evoked GLP-1 and thereby contribute to between-study heterogeneity. In ADDITION-PRO, overweight/obesity and prediabetes or screen-detected type 2 diabetes were associated with smaller oral-glucose GLP-1 responses, whereas higher responses were associated with older age, female sex, greater insulin sensitivity, and better β-cell function [152]. However, a smaller matched study found comparable GLP-1 responses across normal glucose tolerance, impaired glucose tolerance, and mild type 2 diabetes after glucose and mixed meals [153]; another meal study found reduced GLP-1 in obese type 2 diabetes but not obesity alone and demonstrated a meal-size effect [154]. Metabolic phenotype is therefore not a fixed modifier independent of stimulus and design. Baseline gastric emptying controls intestinal nutrient delivery and incretin release [155,156,157], while endogenous GLP-1 can itself slow emptying [149], making gastric emptying both a potential mediator and confounder. Metformin has been associated with higher fasting GLP-1 without a larger 60-min post-meal increment [158]. Fasting concentrations, total versus incremental AUC, medication, insulin sensitivity, age, sex, body composition, and gastric emptying should therefore be reported, and lean, obese, diabetic, and older cohorts should not be pooled without prespecified interaction analyses.

Clinical endpoints provide a more direct basis for evaluating efficacy than hormone concentrations or ratings on visual analogue scales. For acute studies, postprandial glucose and objectively measured ad libitum intake are more informative than hormone concentration alone. Repeated interventions can also examine continuous glucose monitoring, HbA1c, body weight and fat mass, waist circumference, and tolerability. Target engagement, an independent endpoint, and temporal coherence help distinguish a mechanistic GLP-1 effect from a parallel biomarker response. Receptor antagonism or prespecified causal mediation can provide further support, but these approaches are uncommon in food studies.

## 8. Analytical and Study Design Considerations

### 8.1. What Is Being Measured: Total Versus Active GLP-1

The term “GLP-1” is analytically incomplete. L cells predominantly release biologically active GLP-1(7–36)NH_2_, with smaller amounts of GLP-1(7–37). DPP-4 rapidly removes the N-terminal dipeptide, generating GLP-1(9–36)NH_2_ and GLP-1(9–37), which have little classical GLP-1 receptor agonism [11,12,13]. C-terminal (“total GLP-1”) assays generally detect intact peptide plus its primary DPP-4 metabolite and are therefore the more robust proxy for secretion, provided clearance is not altered. N-terminal (“active” or “intact”) assays quantify peptide capable of activating the receptor but reflect secretion, degradation, extraction and clearance simultaneously.

The two measurements are not interchangeable. Commercial assays vary in epitope recognition, cross-reactivity, sensitivity, and matrix effects [159,160,161]. Absolute concentrations from different platforms cannot be assumed to be harmonised. Interpretation is easier when reports identify the assay manufacturer, catalogue number, principle, epitope, molecular forms detected, sample matrix, quantification limit, extraction and recovery, assay precision, dilution linearity, freeze–thaw handling, and batch allocation. Multiplex assays require particular caution at low postprandial concentrations.

Preanalytical handling is critical for active GLP-1. Validated procedures use an anticoagulant tube containing a DPP-4 inhibitor, immediate mixing, cold handling, prompt centrifugation at 4 °C, aliquoting, and storage at −80 °C or below. Processing time is an important reporting detail [13,160]. Aliquoting is prudent to minimise freeze–thaw exposure, although one stability study found no significant GLP-1 change after up to three cycles [160]. “EDTA plasma” alone is insufficient because EDTA does not fully prevent cleavage of the N-terminus by DPP-4 [13]. The inhibitor in the collection tube prevents ex vivo degradation only; it does not show that an ingested food inhibited DPP-4 in vivo. Interpretation of a food as a DPP-4 inhibitor is better supported when total and active GLP-1, the active:total ratio, plasma DPP-4 activity, and exposure to the putative inhibitory peptide or metabolite are considered together. Total GLP-1 is the appropriate primary hormone endpoint when secretion is central. Systemic receptor exposure is better assessed with active GLP-1 measured alongside total GLP-1.

### 8.2. Meal Test Design, Sampling and Gastric Emptying

Acute food interventions are often evaluated in randomised crossover meal tests, which reduce between-participant variability. Interpretation is clearest when test and control meals are comparable in energy, macronutrients, volume, texture, viscosity, palatability, and eating rate, with any remaining differences reported or modelled. These properties alter oral processing, gastric emptying, and intestinal nutrient delivery and can change GLP-1 without a specific molecular action. A water comparator is therefore difficult to interpret when the intervention provides meaningful protein or energy, unless the question concerns the net effect of consuming the product.

The sampling window should be aligned with the expected mechanism, because nutrient-driven responses generally occur earlier than effects requiring colonic fermentation or repeated exposure. Concentration–time curves with prespecified measures such as iAUC, total AUC, Cmax, and Tmax distinguish the magnitude and timing of the response, whereas isolated fasting and postprandial measurements do not adequately characterise GLP-1 secretion.

Measurement of gastric emptying is particularly informative when it forms part of the proposed mechanism or when interventions differ materially in viscosity, structure, energy density, or lipid delivery. Emptying rate determines the timing and magnitude of nutrient exposure in the small intestine and incretin secretion [155,156,157]. Endogenous GLP-1 can itself slow emptying and attenuate glycaemia [149]. Because emptying both drives and responds to GLP-1, it can act as a confounder or mediator. Scintigraphy remains the reference method; validated breath tests using stable isotopes are a pragmatic alternative when their limitations are reported [162,163]. The time to half-emptying measured with a breath test is not interchangeable with scintigraphic emptying. Acetaminophen appearance is an indirect proxy for the early liquid phase, not a validated measure of emptying for a mixed solid meal. Meal sequence, viscosity, particle structure, and gastric retention are better treated as possible mechanisms than as nuisance variables.

### 8.3. Participant Characteristics and Concomitant Measures

Standardising the prior evening meal and activity, duration of fast, test time, sleep, alcohol, caffeine, smoking and recent exercise reduces avoidable variability [164]. Recording sex and menstrual status, age, BMI and body composition, glucose tolerance, baseline gastric emptying and habitual fibre intake helps define the study population. Pooling normal glucose tolerance, prediabetes and type 2 diabetes without prespecified interaction analyses can obscure differences related to metabolic phenotype, age and meal size [152,153,154]. Participants with bariatric surgery or major renal, hepatic, or gastrointestinal disease are often excluded or analysed separately.

Medication is a major confounder. Metformin can raise fasting total and active GLP-1 [158]. DPP-4 inhibitors approximately double intact incretin concentrations without necessarily changing total GLP-1 or gastric emptying [165]. GLP-1 receptor agonists, prokinetics, opioids, and anticholinergic drugs alter the axis directly or through motility. When washout is not ethical, stratification by stable therapy and complete reporting aid interpretation.

Depending on the hypothesis, complementary measures may include glucose, insulin or C-peptide, glucagon, and selected gut hormones. For microbial hypotheses, circulating short-chain fatty acids, bile acids, or metabolites derived from the ingredient provide exposure data, whereas faecal SCFA concentrations do not measure colonic production. Statistical models can account for crossover period and sequence, baseline value, and repeated measurements. Multiplicity correction is relevant when many hormones and time points are explored, and sample size calculations are most appropriately based on the prespecified primary outcome.

## 9. Implications for Functional Food Research

The relevance of a functional food to endogenous GLP-1 depends less on the presence of a candidate bioactive than on whether the finished product changes nutrient or metabolite exposure at the appropriate intestinal site. Chemical characterisation and digestion models can identify plausible ligands and describe their release, but they do not establish an endocrine effect in humans. Acute human studies can relate a formulation to GLP-1 kinetics and independently relevant outcomes, whereas longer interventions provide information about persistence, tolerability, and clinical relevance.

Formulation effects also need to be interpreted at the level of the complete food. Changes in protein structure, cellular integrity, viscosity, or colonic release may alter nutrient delivery, but added protein or lipid energy, sodium, sweeteners, allergenicity, nausea, and bloating can offset a modest incretin response. Product composition, comparator choice, and source of funding therefore remain relevant to interpretation.

Claims are most informative when they correspond to the endpoint that was demonstrated. “Supports postprandial GLP-1 secretion” is reasonable when reproducible human secretion data are available. If improved glycaemia occurs alongside higher GLP-1 but mediation has not been tested, “improves postprandial glycaemia, accompanied by higher GLP-1” is more precise. Labels such as “GLP-1 food”, “natural Ozempic”, and “dietary GLP-1 agonist” conflate transient endogenous secretion with prolonged pharmacological receptor agonism. Dietary modulation of GLP-1 is better understood as one component of a wider postprandial response involving nutrient sensing, gastric emptying, insulin and glucagon secretion, other gut peptides, microbial metabolites, and central appetite control.

## 10. Future Perspectives

Future mechanistic studies should quantify the structural and chemical changes produced by processing rather than rely on ingredient names or broad processing categories. Multiscale characterisation should be paired with time-resolved digestion, measurement of candidate ligands in digesta, and polarised human intestinal models that can distinguish apical from basolateral sensing and proximal from distal responses. Receptor perturbation and measured target exposure are needed before assigning a response to a named pathway.

Human trials should use composition- and energy-matched comparators when the question concerns structure, while retaining water or no-product comparators only when the net effect of consumption is the question. Prespecified GLP-1 forms and kinetic metrics, sufficiently long sampling, validated gastric-emptying measures, and concurrent glucose, insulin, other gut hormones, symptoms, appetite, and intake would make discordant findings interpretable. Stratification or interaction analyses should address metabolic phenotype, age, sex, medication, and baseline gastric emptying.

The most useful candidates are formulations that reproduce a defined exposure profile across batches and independent laboratories, remain acceptable and tolerable at realistic serving sizes, and improve an outcome beyond GLP-1 concentration alone. Longer and independently replicated interventions are required to establish persistence, adaptation, safety, and clinical relevance. Designs should therefore progress from structural characterisation and bioaccessibility to target engagement, acute human physiology, and finally durable metabolic outcomes, rather than selecting products solely by the largest short-term hormone peak.

## 11. Conclusions

Food structure and processing alter the identity, rate, and intestinal location of digestion products that engage enteroendocrine pathways. A reproducible exposure profile is therefore more informative than the largest GLP-1 peak. An early sharp response may accompany rapid nutrient availability, whereas a smaller but prolonged response may reflect delayed or distal delivery. Neither pattern alone predicts appetite, energy intake, or longer-term metabolic benefit. GLP-1 remains one component of a response that also includes gastric emptying, GIP, PYY, CCK, insulin, glucose appearance, gastrointestinal symptoms, and eating behaviour.

Among dietary interventions, whey-containing preloads provide the most consistent evidence for an acute increase in GLP-1 and an improvement in postprandial glycaemia. Even in this setting, effects on appetite, energy intake, and body weight are inconsistent. Findings for fibres, lipid emulsions, polyphenols, bitter compounds, fermented foods, and controlled-release formulations depend more strongly on the material, dose, delivery route, matrix, and population. Glycaemia or gastric emptying can also improve without an increase in GLP-1, which limits the use of the hormone as a general marker of food efficacy.

Structural characterisation, digestion models, enteroendocrine cell systems, and human studies address different parts of this question. Standardised static digestion can describe initial release under controlled conditions [38,39], whereas dynamic models and time-resolved measurements provide additional information about persistence and distal delivery. Conventional cell lines remain useful for screening, although their peptide repertoire differs from that of native L cells [166]. In human studies, GLP-1 profiles are most informative when considered alongside gastric emptying, glycaemia, other gut hormones, symptoms, and food intake.

Engineered food architecture, thermal starch treatment, leavening, oat processing, and soy-protein extraction have been associated with altered GLP-1 responses; however, these hormonal changes were not consistently accompanied by changes in glycaemia, satiety, or energy intake [92,97,108,138,139].

Overall, current evidence supports modest and context-dependent dietary modulation of endogenous GLP-1, not food-based reproduction of pharmacological receptor agonism. Food-derived secretagogues, DPP-4 inhibitors, delivery systems, and direct receptor agonists are mechanistically distinct. The translational relevance of a formulation ultimately depends on a reproducible and independently meaningful physiological outcome, not on GLP-1 concentration alone.

## Figures and Tables

**Figure 1:**
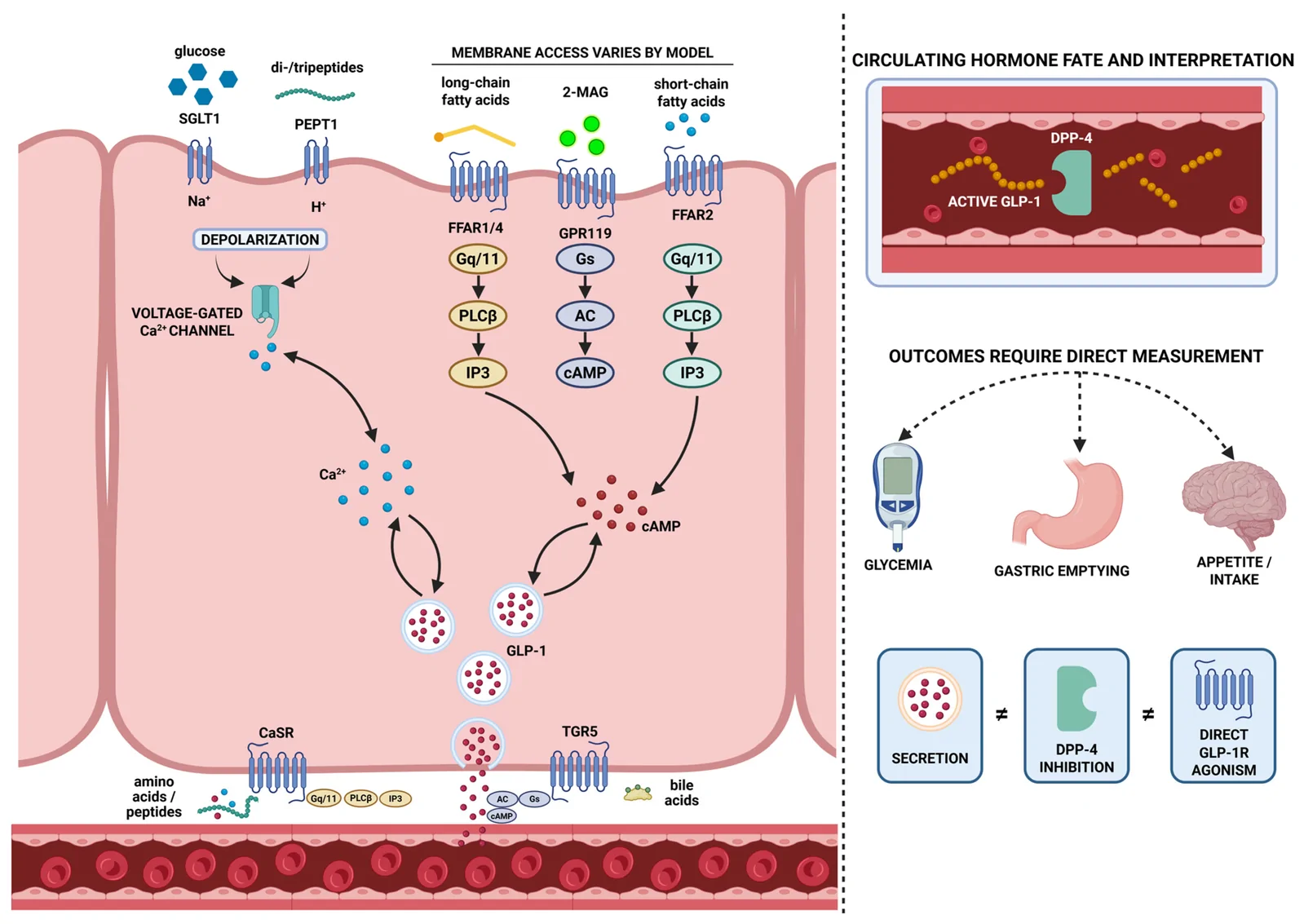
Food-derived nutrient sensing, endogenous GLP-1 secretion, and interpretation of circulating responses. SGLT1-mediated glucose uptake and PEPT1-mediated di- and tripeptide uptake depolarise the enteroendocrine membrane and promote Ca^2+^ influx through voltage-gated channels. Long-chain fatty acids, selected 2-monoacylglycerols, short-chain fatty acids, amino acids or peptide digests, and bile acids engage FFAR1/4, GPR119, FFAR2, CaSR, and GPBAR1/TGR5, respectively. Gq/11-PLCβ-IP_3_ signalling increases intracellular Ca^2+^, whereas Gs-AC signalling increases cAMP; both pathways converge on GLP-1 granule exocytosis. Sensor localisation and membrane access vary by intestinal region, species, epithelial polarity, and experimental model, so the depicted sidedness is schematic. After secretion, active GLP-1 is rapidly cleaved by DPP-4, and its circulating concentration therefore reflects secretion, degradation, distribution, and clearance. Endogenous secretion, DPP-4 inhibition, and direct GLP-1R agonism are mechanistically distinct. Glycaemia, gastric emptying, appetite, and energy intake must be measured independently rather than inferred from circulating GLP-1 alone. The schematic is simplified and non-exhaustive. Abbreviations: 2-MAG, 2-monoacylglycerol; AC, adenylyl cyclase; cAMP, cyclic adenosine monophosphate; CaSR, calcium-sensing receptor; DPP-4, dipeptidyl peptidase-4; FFAR, free fatty acid receptor; GLP-1, glucagon-like peptide-1; GLP-1R, glucagon-like peptide-1 receptor; GPBAR1/TGR5, G protein-coupled bile acid receptor 1/Takeda G protein-coupled receptor 5; GPR119, G protein-coupled receptor 119; Gq/11, Gq/11 family of heterotrimeric G proteins; Gs, stimulatory G protein; IP_3_, inositol 1,4,5-trisphosphate; PEPT1, peptide transporter 1; PLCβ, phospholipase C beta; SGLT1, sodium–glucose cotransporter 1. Created in https://BioRender.com.

**Table 1:** (**A**) Nutrient-derived ligands and intestinal sensing routes that modify endogenous GLP-1 secretion. (**B**) Microbial and phytochemical pathways and other proposed routes affecting endogenous GLP-1 physiology.

(**A**)				
**Dietary Input or Ligand Class**	**Exposure-Defining Chemical Features**	**Principal Sensor or Mechanism**	**Representative Evidence**	**Key Limitation**
Hexoses transported by SGLT1 (glucose and galactose)	Monosaccharide identity, absorption rate, luminal concentration, and site of release	Electrogenic SGLT1 uptake; depolarization and Ca2+ entry	Dominant SGLT1 dependence in the human small intestine and models with experimental SGLT1 manipulation [29,48,49]	Sweetness is not equivalent to transport; non-nutritive sweetener effects are inconsistent [51,115]
Amino acids and oligopeptides	Sequence, charge, hydrophobicity, peptide length, digestive stability, and membrane side	PEPT1 uptake, CaSR, amino acid transport and metabolism; GPR142 remains an emerging candidate	One human organoid study with knockout of multiple receptors supports CaSR; postprandial responses to whey are reproducible [40,46,62]	No active peptide and receptor pair has been established in humans [64,66]
Long-chain fatty acids	Chain length, unsaturation, ionization, free versus esterified state, and lipolysis rate	FFAR1/GPR40 and FFAR4/GPR120; predominantly Gq/Ca2+ signalling	Human evidence for effects of chain length is complemented by FFAR4 results from the same human organoid atlas [40,70,71]	Energy load, nausea, and gastric emptying confound clinical interpretation
2-Monoacylglycerols	sn-2 acyl identity and quantity generated during triacylglycerol digestion	GPR119 signalling through Gs and cAMP	2-Oleoylglycerol activates GPR119 and raises GLP-1 in humans [72,73]	Evidence derives mainly from small acute studies; efficacy over longer periods is untested
(**B**)				
**Dietary Input or Ligand Class**	**Exposure-Defining Chemical Features**	**Principal Sensor or Mechanism**	**Representative Evidence**	**Key Limitation**
SCFAs from fermentable substrates	Polymer linkage, fermentability, production rate, location, absorption, and SCFA species	FFAR2/FFAR3 plus metabolism independent of these receptors and HDAC effects	One programme targeting propionate delivery to the colon provides human proof of concept [31,109]	Ordinary fibres are heterogeneous; receptor causality and faecal SCFA interpretation are uncertain [30]
Primary and secondary bile acids	Hydroxylation, conjugation, microbial conversion, epithelial transport, and access to the apical or basolateral surface	GPBAR1/TGR5 stimulates secretion; intestinal FXR may oppose GLP-1 production	Basolateral TGR5 sensing plus GPBAR1 knockout in the same human organoid atlas [40,47,76]	A dietary intervention rarely isolates a defined bile acid species from transit and microbiome effects
Bitter phytochemicals and tastants	Aglycone/glycoside form, subtype selectivity, solubility, metabolism, and achievable luminal dose	TAS2R signalling through gustducin, PLC, and Ca2+; other pathways frequently coexist	Human quinine proof of principle; mainly cell/animal data for named phytochemicals [77,79,81]	Culinary exposure is not equivalent to intragastric/intraduodenal pharmacological dosing
Food peptides that inhibit DPP-4	Sequence, gastrointestinal survival, epithelial permeability, free concentration, and target engagement	Preservation of intact GLP-1; not secretion from L cells	Extensive activity in cell-free assays but limited validation in humans [68,69]	Interpretation depends on total and active GLP-1, their ratio, plasma DPP-4 activity, and peptide exposure
Purported food compounds acting directly as GLP-1R agonists	Receptor binding, GLP-1R activation measured through cAMP and β-arrestin signalling, exposure, and pharmacokinetics	Direct GLP-1R activation, conceptually separate from endogenous secretion	No convincing evidence of target engagement in humans at a dose achievable from food; plant modulators remain preclinical [116]	Docking, glucose lowering, or a rise in plasma GLP-1 does not demonstrate receptor agonism

Abbreviations: CaSR, calcium-sensing receptor; DPP-4, dipeptidyl peptidase-4; FFAR, free fatty acid receptor; GPBAR1, G-protein-coupled bile acid receptor 1; HDAC, histone deacetylase; PEPT1, peptide transporter 1; SCFA, short-chain fatty acid; SGLT1, sodium–glucose cotransporter 1; TAS2R, type 2 taste receptor.

**Table 2:** Processing and formulation operations that modify food structure and alter digestion kinetics or the intestinal delivery of L-cell ligands.

Processing or Formulation Operation	Structural Feature Modified	Consequence for Digestion or Delivery	Representative Evidence	Key Limitation
Milling, fractionation, and cell rupture	Cell wall continuity, particle size distribution, porosity, damaged starch	Changes the area accessible to enzymes, pyloric sieving, and timing of sugar and peptide release	Three related chickpea studies found delayed release or longer GLP-1/PYY profiles with intact cells or coarser structure [17,89,90]	The comparison involving coarse bread was not matched for composition; cell integrity and structural persistence are more informative than sieve size alone
Thickening, gelation, and hydrothermal treatment	Viscosity at gastric pH, gel strength, gelatinization, crystallinity, retrogradation	Modulates mixing, emptying, diffusion, and amylolysis; may flatten rather than increase hormone exposure	The oat beverage with lower viscosity increased early GLP-1. The beverage with higher viscosity delayed emptying without improving PYY or appetite, while β-glucan bread delayed emptying without changing GLP-1 [18,19,93]	Early and late sampling windows help distinguish redistribution from a change in overall exposure
Homogenization and emulsification	Droplet size, interface, acid stability, coalescence, creaming, solid fat content	Controls gastric separation, interfacial area, lipolysis, and delivery of fatty acids and 2-MAG	Acid stability and lipid physical state altered emptying, satiety, and GLP-1 in factorial human studies [20,98,99]	Characterisation after oral and gastric challenge is more informative than manufacturing data alone
Heating, mineral addition, and enzymatic proteolysis	Unfolding, aggregation, mineral bridging, solubility, degree of hydrolysis, peptide size distribution	Alters gastric structuring, protease access, peptide release, taste, and absorption	Calcium effects depended on β-lactoglobulin conformation; intact and hydrolysed proteins often gave similar GLP-1 [100,101,104]	Combined structural data and digestion peptidomics improve interpretation; degree of hydrolysis is not a mechanism
Carbohydrate redesign	Amylose association, resistant fraction, glycosidic linkage, slowly available carbohydrate	Redistributes glucose delivery between proximal sensing and later fermentation	A resistant-starch/pullulan combination reduced glucose and GLP-1 acutely, while other studies of resistant starch depended on phenotype [94,95,117]	Digestible fractions in the prepared food are more informative than ingredient specifications; slow release need not increase GLP-1
Encapsulation and targeted intestinal release	Payload, loading, coating chemistry, response to pH and enzymes, recovery, and mass balance	Moves nutrients or precursors to ileum/colon and prolongs local exposure	Ileal infusion showed positional effects; one propionate programme was positive, while enteric glutamine was weak [33,34,109,110]	Endocrine attribution depends on the release site and recovery of the payload

Abbreviations: 2-MAG, 2-monoacylglycerol; GLP-1, glucagon-like peptide-1; PYY, peptide YY.

**Table 3:** Comparator design and concordance between GLP-1 responses and independent outcomes in human food-intervention studies.

Intervention	Population	Comparator Type *	GLP-1 Finding	Independent Endpoint(s)	Key Limitation
Whole meals, dietary patterns, and meal order [128,129,130,131]	Healthy, obese, and T2D adults; postmenopausal women with obesity	M: [128,131]; P: [129]; A: [130]	Higher after vegan [128], Mediterranean [129], and fish/meat-first meals [131]; oral-glucose iAUC increased after weight loss in both long-term diets [130]	Satiety increased [128]; glucose decreased without appetite change [129]; glucose and emptying changed together [131]; weight loss occurred in both groups [130]	Mostly small acute trials; composition and emptying co-varied; weight loss confounded [130]
Whey/protein preloads [63,132,133,134]	Healthy young men and adults with T2D, including those with overweight or obesity	M + W: [63]; P + W: [132]; W: [133,134]	Higher with whey [63,132]; lower postprandial concentrations at weeks 1/12 [133]; total GLP-1 0–2 h iAUC increased 66% [134]	Glucose decreased and emptying slowed [63,132]; HbA1c fell 0.1 percentage point without weight/intake change [133]; glucose iAUC fell 22% while insulin rose [134]	Water/no-whey contrasts were not energy matched; insulin and emptying are co-mediators; product-specific evidence
Protein physical form/formulation [100,135]	Healthy adults	M/P: [100]; P + W: [135]	Total GLP-1 iAUC depended on β-lactoglobulin form × calcium [100] and on protein dose/formulation [135]	Emptying changed but intake did not clearly fall [100]; total intake was higher after caloric drinks than control [135]	Small acute studies; [135] used isoenergetic or protein-matched drinks, with other macronutrients differing
Fermentable and novel fibres [31,94,136,137]	Healthy, lean, overweight, and obese adults	P: [31]; M: [94]; A: [136,137]	Increased with targeted propionate [31]; decreased with resistant starch + pullulan [94]; unchanged after acute inulin/resistant starch [136]; no between-group difference; within-group increase only with cellulose [137]	Intake/weight gain attenuated [31]; metabolic benefit without satiety [94]; no weight/adiposity effect [137]	Fibre chemistry, carrier, dose, and sampling/adaptation period differed
Grain, pulse, and bread structure [17,19,89,138,139]	Mainly healthy adults	P: [19,89]; M: [17]; A: [138,139]	No change [19]; early versus prolonged profiles by chickpea structure [17]; increased/sustained with cellular flour [89]; modest leavening differences [138]; lower iAUC with some oat products [139]	Glycaemia often improved, whereas appetite, emptying, or intake were inconsistent	Solid/liquid matrices, doses, and processes differed; [17,89] were not independent replications
Lipid-derived ligands [71,140,141]	Healthy adults and adults with T1D	W + M: [71]; P/A: [140,141]	C12 and olive oil increased GLP-1 relative to controls/comparator fats	C12 reduced intake but caused nausea [71]; extra-virgin olive oil lowered glycaemia and altered emptying [141]	Intraduodenal delivery, high energy loads, nausea, and very small samples
Lipid emulsions [20]	Healthy adult men (n = 15)	P: [20]	Liquid acid-stable emulsion increased 6-h GLP-1 iAUC by 38% versus solid acid-unstable and by 76% versus liquid acid-unstable emulsions	Acid-stable emulsions delayed gastric emptying; the liquid acid-stable formulation reduced hunger and desire to eat versus the solid acid-unstable formulation	Small acute trial in men; lipid fractions and emulsifiers differed; GLP-1 mediation untested
Polyphenols and bitter/tastant compounds [79,142,143,144]	Healthy adults and older men with T2D	A: [142]; capsule placebo: [143]; W: [79,144]	Cinnamon increased ΔAUC/Cmax [142]; oleuropein increased 2-h GLP-1 [143]; quinine increased postdrink GLP-1 at 600 mg but not 300 mg [79]; tastants caused no change [144]	Glycaemia improved in some studies, but intake was unchanged with quinine and fell without hormone change after mixed tastants	Acute small trials; intraduodenal dosing is not food-realistic; mediation untested
Probiotics and fortified yogurt [145,146]	Glucose-tolerant lean/obese adults and adults with obesity during energy restriction	Caplet placebo: [145]; A: [146]	Oral-glucose-stimulated GLP-1 increased with L. reuteri [145]; fasting GLP-1 differed between yogurt groups [146]	Insulin secretion increased without better sensitivity/body mass [145]; independent clinical effects remained unclear [146]	Strain/product specificity, concurrent energy restriction, and fasting rather than postprandial measurement

* M, energy- and macronutrient- or iso-nutrient-matched comparator (including the same foods in a different order); P, partially matched comparator; W, water or negligible-energy vehicle; A, other active comparator. AUC, area under the curve; GLP-1, glucagon-like peptide-1; iAUC, incremental AUC; T1D, type 1 diabetes; T2D, type 2 diabetes.

## Data Availability

No new data were created or analyzed in this study. Data sharing is not applicable to this article.

## Abbreviations

The following abbreviations are used in this manuscript:

CaSR	calcium-sensing receptor
CCK	cholecystokinin
DPP-4	dipeptidyl peptidase-4
FFAR	free fatty acid receptor
FXR	farnesoid X receptor
GIP	glucose-dependent insulinotropic polypeptide
GLP-1	glucagon-like peptide-1
GLP-1R	glucagon-like peptide-1 receptor
GPBAR1/TGR5	G protein-coupled bile acid receptor 1, also known as Takeda G protein-coupled receptor 5
GPR119	G protein-coupled receptor 119
PEPT1	peptide transporter 1
PYY	peptide YY
SGLT1	sodium–glucose cotransporter 1
TAS2R	taste receptor type 2
